# A Developmental Stage-Specific Switch from DAZL to BOLL Occurs during Fetal Oogenesis in Humans, but Not Mice

**DOI:** 10.1371/journal.pone.0073996

**Published:** 2013-09-25

**Authors:** Jing He, Kayleigh Stewart, Hazel L. Kinnell, Richard A. Anderson, Andrew J. Childs

**Affiliations:** 1 MRC Centre for Reproductive Health, the Queen’s Medical Research Institute, University of Edinburgh, Edinburgh, United Kingdom; 2 Department of Comparative Biomedical Sciences, the Royal Veterinary College, University of London, Camden, London, United Kingdom; University Hospital of Münster, Germany

## Abstract

The *Deleted in Azoospermia* gene family encodes three germ cell-specific RNA-binding proteins (DAZ, DAZL and BOLL) that are essential for gametogenesis in diverse species. Targeted disruption of *Boll* in mice causes male-specific spermiogenic defects, but females are apparently fertile. Overexpression of human BOLL promotes the derivation of germ cell-like cells from genetically female (XX), but not male (XY) human ES cells however, suggesting a functional role for BOLL in regulating female gametogenesis in humans. Whether BOLL is expressed during oogenesis in mammals also remains unclear. We have therefore investigated the expression of BOLL during fetal oogenesis in humans and mice. We demonstrate that BOLL protein is expressed in the germ cells of the human fetal ovary, at a later developmental stage than, and almost mutually-exclusive to, the expression of DAZL. Strikingly, BOLL is downregulated, and DAZL re-expressed, as primordial follicles form, revealing BOLL expression to be restricted to a narrow window during fetal oogenesis. By quantifying the extent of co-expression of DAZL and BOLL with markers of meiosis, we show that this window likely corresponds to the later stages of meiotic prophase I. Finally, we demonstrate that Boll is also transiently expressed during oogenesis in the fetal mouse ovary, but is simultaneously co-expressed within the same germ cells as Dazl. These data reveal significant similarities and differences between the expression of *BOLL* homologues during oogenesis in humans and mice, and raise questions as to the validity of the *Boll^-/-^* mouse as a model for understanding BOLL function during human oogenesis.

## Introduction

The *Deleted in Azoospermia* (*DAZ*) gene family encodes three conserved RNA-binding proteins (DAZ, DAZL and BOLL), the expression of which is largely restricted to germ cells. DAZ-family proteins are essential for germ cell development in diverse organisms, and inactivating mutations in members of the DAZ family result in a failure of germ cell development in either or both sexes [1-4]. DAZ-family proteins are characterised by a conserved RRM-type RNA binding domain, and a unique 24 amino-acid DAZ repeat sequence, and act as regulators of mRNA translation [5], mRNA transport [6] and mRNA metabolism during cellular stress [7]. The sterility phenotypes observed in DAZ-family protein-deficient organisms are believed to arise from a failure to translate key mRNAs required for germ cell development [5,8-11].

The prototypic *DAZ* gene is present in multiple copies on the Y chromosome of humans and Old World monkeys [12], and was identified as a candidate male factor infertility gene from its location in the *AZFc* region of the Y chromosome, an area frequently deleted in men with severe oligozoospermia or azoospermia [13]. *DAZ* arose from a duplication of the autosomal homologue *Dazl* (*Deleted in azoospermia-like*) [14], the expression of which is required at multiple stages of germ cell development in male and female mammals [1,11,15-20]. *BOLL* (also known as *BOULE*, or *BOULE-LIKE*), is the ancestral member of the family, with orthologues throughout the metazoa [21,22]. *Boule*-deficient flies display defective spermatogenesis [3], arising from a failure to translate mRNA encoding the CDC25 homologue Twine, resulting in meiotic arrest [8]. Targeted disruption of *Boll* in mice results in male-specific infertility [22], due to arrest of spermiogenesis at the round spermatid stage [4]. *Boll-*deficient female mice are apparently fertile however, leading to the hypothesis that Boll is dispensable for mammalian oogenesis [4,22]. Intriguingly however, overexpression of human BOLL can enhance the derivation of primordial germ cell-like cells from genetically female (XX) but not male (XY) human embryonic stem cells [23], suggesting a potential functional role for BOLL in human female germ cell development that remains to be established.

Although *Boll* transcripts have been reported in the mammalian fetal ovary [22,24,25] the existence of Boll protein in mammalian female germ cells has not yet been demonstrated, and it has been suggested that Boll protein may never be produced in the mammalian female germline [25]. We have previously observed a period of human female germ cell development, prior to primordial follicle formation, during which DAZL expression was reduced [26], and have therefore undertaken the first detailed study of BOLL expression during oogenesis in the fetal mammalian (human and mouse) ovary, and compared this with the expression of DAZL. We demonstrate for the first time that BOLL protein is expressed in the human female fetal germline, and that the expression of DAZL and BOLL is largely non-overlapping, with each protein expressed in a distinct population of germ cells at different developmental stages. Additionally, we show that Boll is also transiently expressed in the germ cells of the fetal mouse ovary, but in striking contrast to the human, shows extensive co-expression in the same germ cells as Dazl. Together, these data indicate that BOLL may have specific and important functions in regulating oogenesis in humans - distinct from those of DAZL - in contrast to its dispensability in mice.

## Results

### DAZL and BOLL show overlapping, but distinct, patterns of gene expression during human fetal ovary development

**Figure 1:**
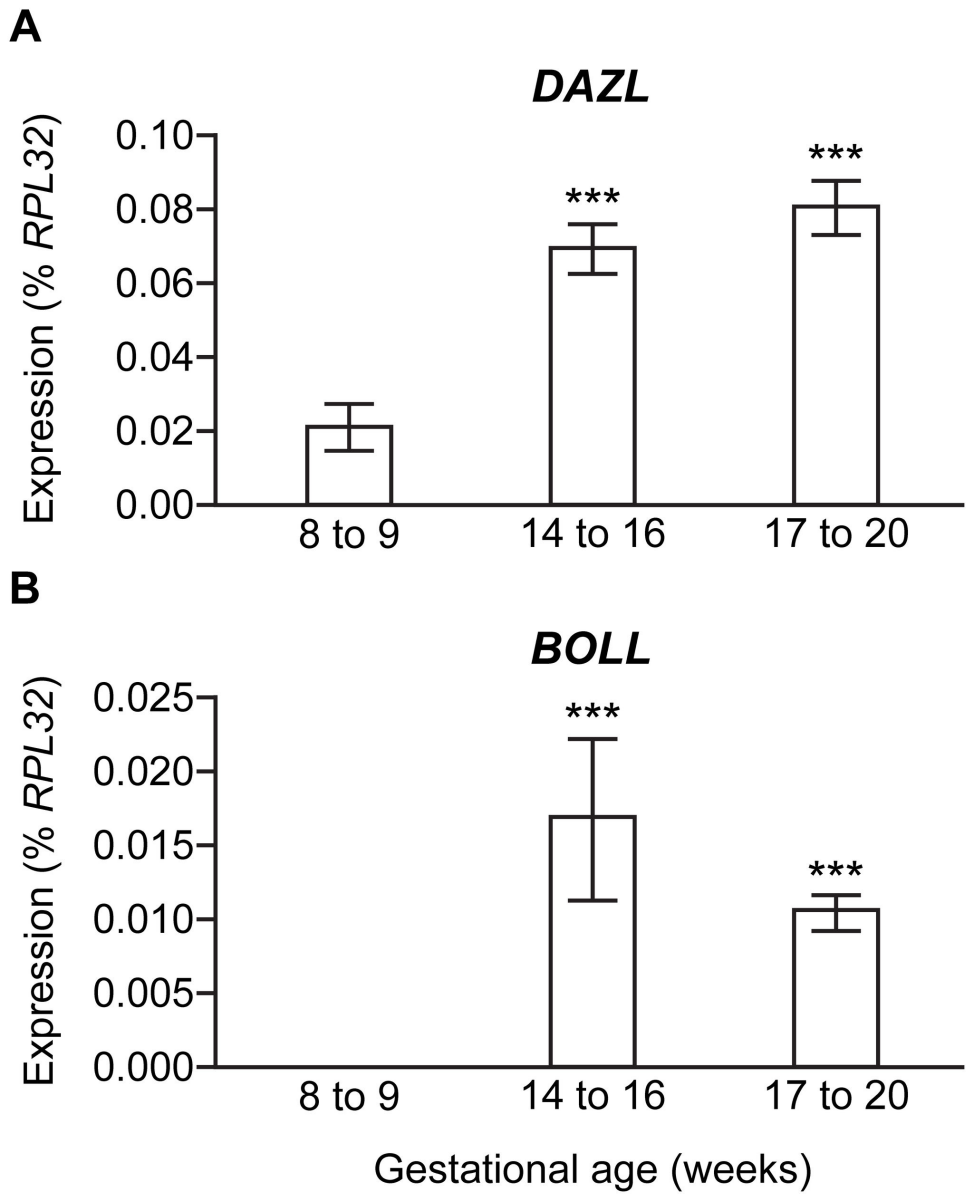
Distinct patterns of *DAZL* and *BOLL* expression during human ovary development. **A**) *DAZL* transcript levels increase significantly from 8-9 to 14-16 weeks, with no significant change thereafter. **B**) *BOLL* expression is first detected at 14-16 weeks gestation, and but does not change significantly by 17-20 weeks (n=5-6, ***p<0.001 vs 8-9 weeks for both DAZL and BOLL).

We first investigated the expression of *DAZL* and *BOLL* during human fetal ovary development, using qRT-PCR (Figure 1A, B). Previous work revealed *DAZL* to be upregulated between the first and second trimesters of human fetal ovarian development [26], however the second trimester encompasses two key developmental stages; the formation of germ cell nests and entry into and progression through meiotic prophase I (approximately 12-16 weeks gestational age (wga)) and the breakdown of germ cell nests and the assembly of individual oocytes into primordial follicles (from around 17wga onwards). We therefore sought to establish at which of these developmental stages the increase in *DAZL* occurs, by dividing the second trimester into early (14-16wga) and late (17-20wga), and compare this to the expression of *BOLL*. In the first trimester (8-9wga), *DAZL* expression is low and *BOLL* undetectable. In the early second trimester (14-16wga) both *DAZL* expression significantly increases, and *BOLL* mRNA first becomes detectable (p<0.001). At both 14-16 and 17-20wga weeks *BOLL* mRNA levels were lower than that of *DAZL*, relative to the internal housekeeping control *RPL32*. Consistent with previous immunohistochemical studies [21] we were unable to detect *BOLL* mRNA in the human fetal testis across this developmental window (data not shown).

### Distinct spatio-temporal distributions of *DAZL* and *BOLL* proteins during human fetal oogenesis

The expression and distribution of DAZL protein in the human fetal ovary has been reported previously [26]. However the expression of BOLL protein in the mammalian ovary has not previously been reported, and it has been proposed that it is not expressed [25], despite the presence of transcript [22,24]. We therefore sought to establish whether BOLL protein is expressed in the human fetal ovary, and if so, what relationship it has to the expression of DAZL.

We confirmed the specificity of the DAZL and BOLL antibodies used in this study (listed in Table S3) by performing immunofluorescence on HEK293 cells transfected with vectors expression either Myc- and FLAG-tagged human DAZL or BOLL (Figure S1). Anti-DAZL antibodies bound epitopes only in cells transfected with DAZL expression constructs, and not in untransfected cells or cells transfected with a BOLL expression vector. Conversely, the anti-BOLL antibody detected signals in BOLL-expressing cells, but not in cells ectopically-expressing DAZL or untransfected controls. We further validated the specificity of the antibodies by performing western blotting on lysates of DAZL and BOLL-transfected HEK293 cells. The anti-DAZL antibodies detected bands of appropriate sizes only in lysates of DAZL-expressing HEK293 cells, and not in those of BOLL-transfected or untransfected cells (Figure S2A,B). Similarly, the anti-BOLL antibody detected a band only in extracts of BOLL-transfected cells (Figure S2C).

**Figure 2:**
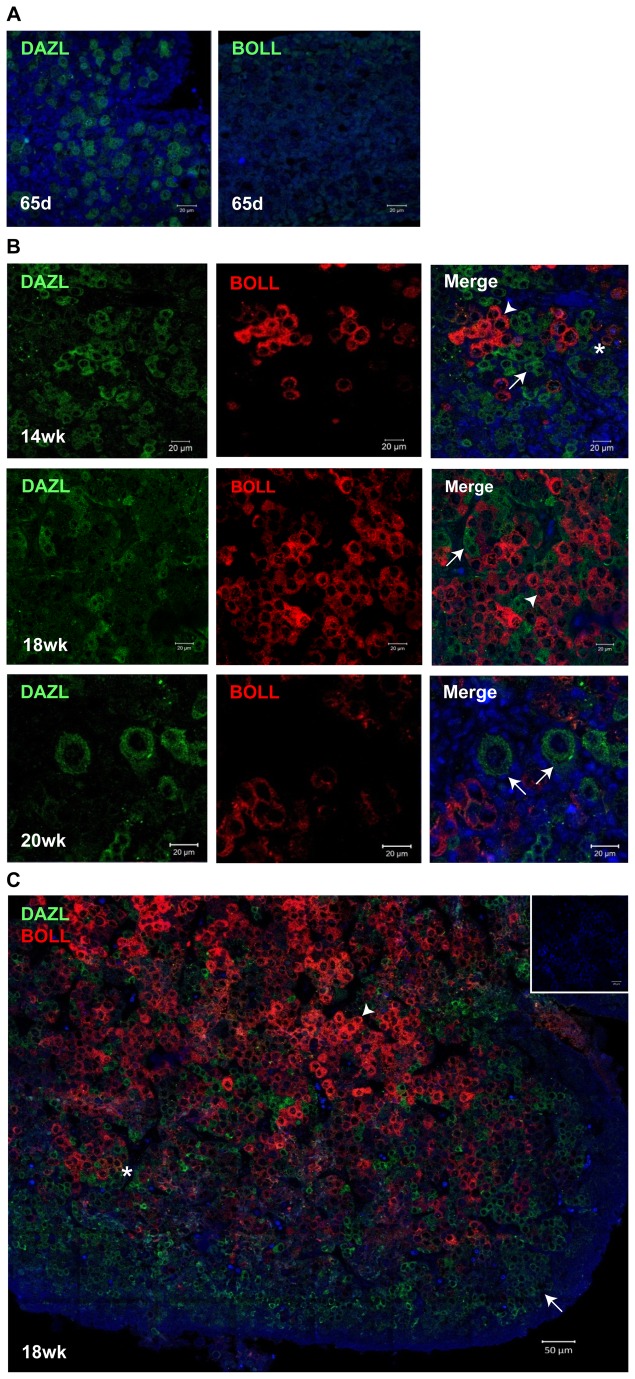
Dynamic changes of DAZL and BOLL protein expression during development of the human fetal ovary. (**A**) at 65d gestation, DAZL is expressed in the nuclei and cytoplasm of germ cells in the human fetal ovary, but BOLL is not detected (**B**) At 14, 18 and 20 weeks gestation, DAZL is detected only in germ cell cytoplasm (arrows). BOLL-positive germ cells (arrowheads) are detectable from 14 weeks onwards, and increase in abundance with increasing gestation. Rare double-positive cells are marked by the asterisk. Primordial follicles at 20 weeks gestation (arrows, bottom left panel) express only DAZL. **C**) Tiled image of 18 weeks gestation fetal ovary section showing minimal co-localisation of DAZL and BOLL. DAZL is expressed in less mature germ cells in a more peripheral localisation, while BOLL predominantly expressed in more mature, centrally-located germ cells. Scale bar (A) and (B) 20 µm, (C) 50µm.

Using single immunofluorescence we were able to detect DAZL protein in the germ cells of 8-9wga human fetal ovaries, with the protein localising to both germ cell nuclei and cytoplasm as reported previously [26,27]. We were unable to detect BOLL protein, consistent with our inability to detect *BOLL* mRNA at this stage (Figure 2A). Both DAZL and BOLL were expressed in germ cells in the second trimester, and dual immunofluorescence analysis revealed that their expression patterns are distinct and dynamic (Figure 2B). At 14wga, most germ cells were DAZL-positive, with relatively few expressing BOLL. By 18wga, BOLL-positive cells were more abundant, although DAZL-positive germ cells were still readily detectable. Notably, we found that the oocytes of primordial follicles at 20wga expressed DAZL, but not BOLL, suggesting oocytes downregulate BOLL and re-express DAZL at or around the time of follicle formation. In all specimens examined, only few germ cells showed expression of both DAZL and BOLL (Figure 2B and C).

We noted distinct spatial distributions of DAZL and BOLL in the human fetal ovary. Consistent with earlier studies [26], we found DAZL-expressing germ cells to be predominantly localised towards the periphery of the ovary, the site of less mature germ cells (Figure 2C). In contrast, BOLL-positive germ cells were localised more towards the central medullary region, which contains the more mature germ cells, and where primordial follicles are first formed. Germ cells expressing both DAZL and BOLL were rare (Figure 2B and C), suggesting DAZL and BOLL mark distinct populations of germ cells. Together, these data reveal that BOLL is indeed expressed in germ cells of the human fetal ovary, and at a later stage of germ cell development than DAZL. Furthermore, the expression of BOLL in germ cells prior to, but not following, follicle formation suggests that BOLL is expressed in a narrow developmental window, subsequent to DAZL expression in early, less mature germ cells, and before DAZL expression is reactivated in primordial follicles.

### *BOLL* is expressed by germ cells at a later stage of development than *DAZL*

**Figure 3:**
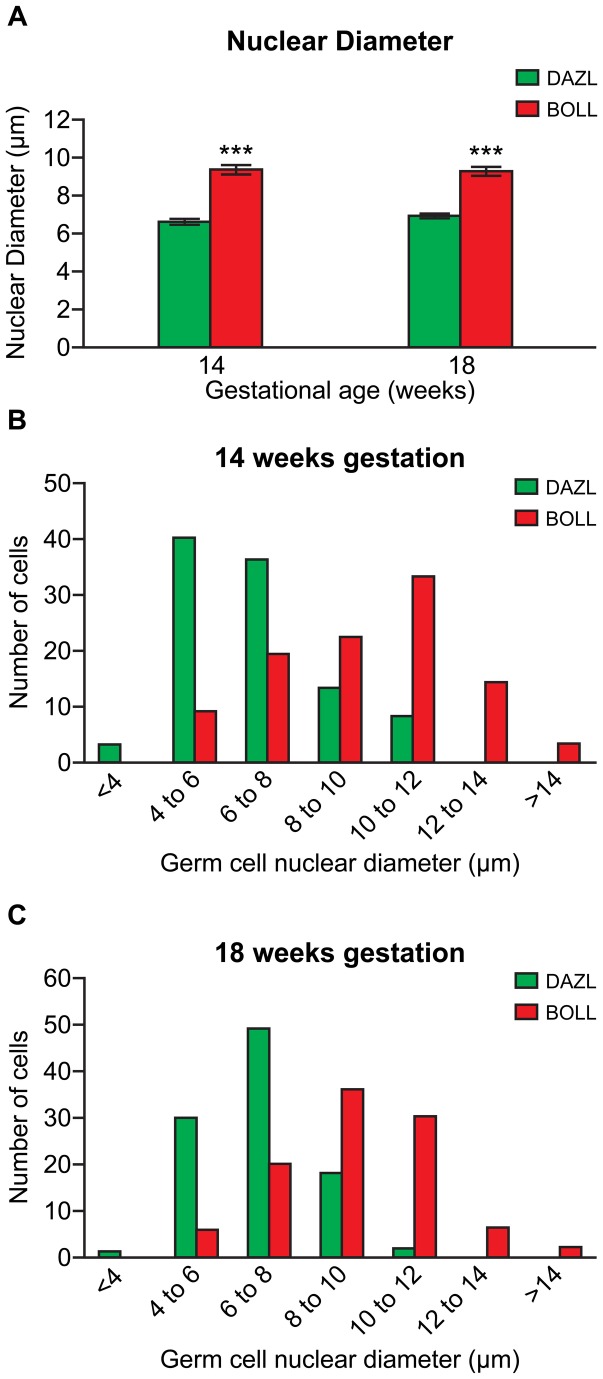
BOLL-positive germ cells are at a later developmental stage than those expressing DAZL. **A**) At 14 and 18 weeks gestation, the average nuclear diameter (an index of germ cell maturation) of BOLL^+^ cells is larger than that of DAZL^+^ cells. **B**) **and** **C**) The nuclear diameter distribution of DAZL and BOLL^+^ cells at 14 and 18 weeks gestation respectively showing distinct size distributions for the two proteins. Primordial follicles were excluded from this analysis (n=108-191 germ cells, ***p<0.001)..

The above data led us to hypothesise that BOLL is expressed in human fetal ovarian germ cells that have reached a later stage of development than those expressing DAZL. To test this, we compared the nuclear diameters of germ cells in the second trimester human ovary that expressed either DAZL or BOLL. Germ cell nuclear diameter provides an index of germ cell maturation, increasing as germ cells progress through development in the human fetal ovary [28]. We found the average nuclear diameter of BOLL-positive germ cells to be significantly greater than that of DAZL-positive germ cells at 14wga (6.60+/-0.12µm vs 9.37+/-0.20µm; p<0.001) and 18wga (6.87+/-0.12µm vs 9.23+/-0.19µm; p<0.001, n=108-191 per group; Figure 3A). Furthermore, DAZL- and BOLL-positive germ cells displayed distinct, although overlapping, nuclear diameter frequency distributions (Figure 3B and C); with most DAZL-positive germ cells distributed between at 4 to 8µm, compared to 8 to 12µm for BOLL-positive germ cells (DAZL-positive oocytes in primordial follicles were excluded from this analysis). Together, these data confirm that BOLL is expressed by germ cells at a later stage of development than those expressing DAZL.

### *DAZL* and *BOLL* differentially associate with markers of meiosis

**Figure 4:**
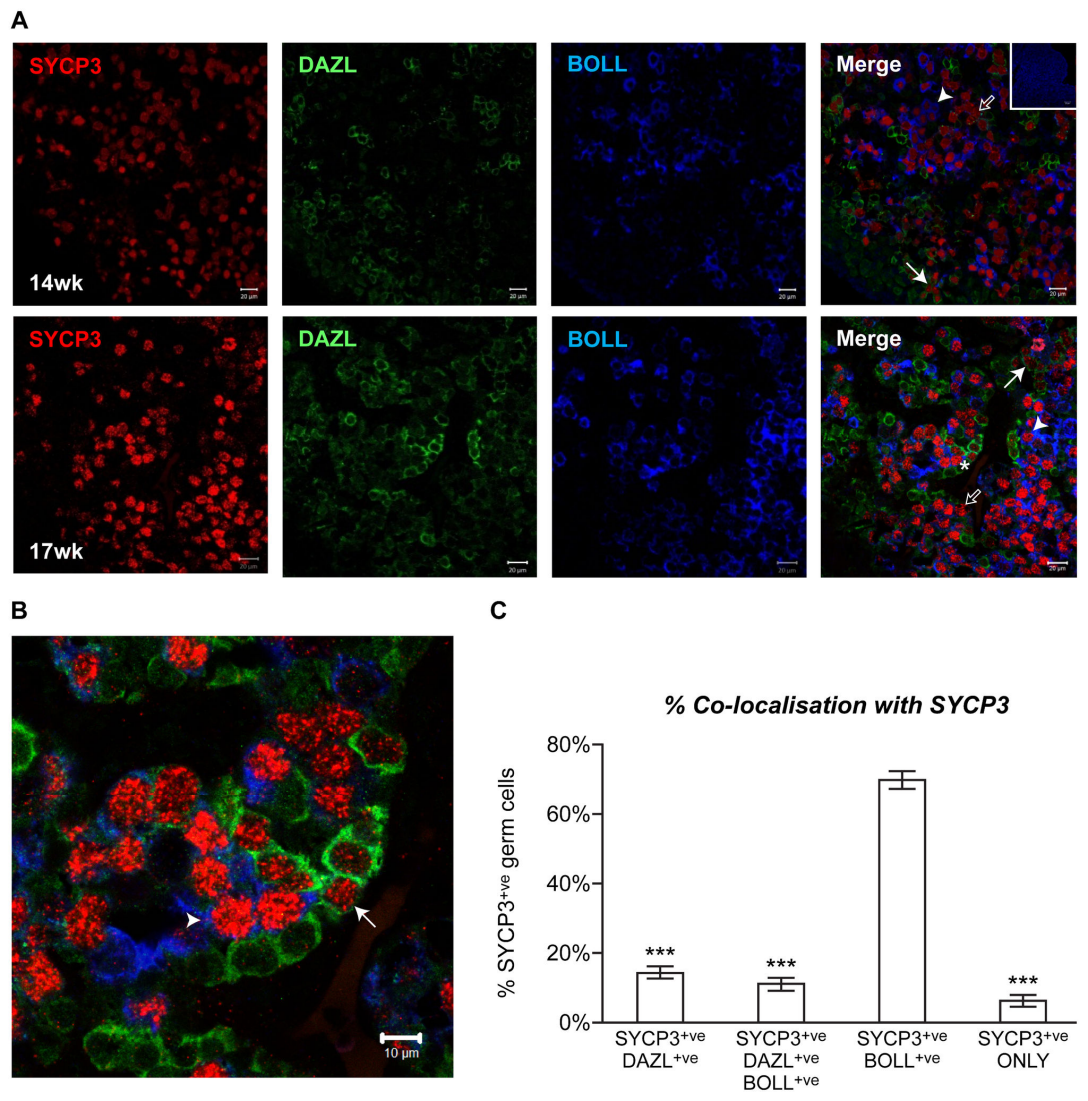
BOLL displays greater co-localisation with the meiosis marker SYCP3 than DAZL. **A**) Triple immunofluorescence analysis of DAZL (green), BOLL (blue) and SYCP3 (red) in 14 and 17 week ovary. All BOLL^+^ germ cells also express SYCP3 (arrowheads), but only a few DAZL^+^ germ cell express SYCP3 (arrows). Unfilled arrows indicate the SYCP3-positive cells that express neither DAZL nor BOLL. The asterisk indicates a germ cell nest containing BOLL ^+^ SYCP3^+^, DAZL ^+^ SYCP3^+^ and DAZL+SYCP- germ cells in close proximity. Scale bars: 20µm. **B**) magnified image of germ cell nest marked with asterisk in A, showing neighboring germ cells expressing different combinations of DAZL, BOLL and SYCP3 expression; arrow denotes DAZL ^+^ SYCP3^+^ germ cell, arrowhead denotes BOLL ^+^ SYCP^+^ germ cell. **C**) Quantification of DAZL, BOLL and SYCP3 co-expression. ~69% of SYCP3^+^ cells also express BOLL^+^. This is significantly greater than the percentage of all the other co-expression patterns (n=3 14-17 week human fetal ovaries; ***p<0.001)..

Between 11wga and 20wga, germ cells in the fetal ovary form syncytial nests and enter the first meiotic prophase, arresting at the diplotene stage (at which they remain until immediately before ovulation). To establish if the timing of the switch from DAZL to BOLL during human fetal oogenesis occurs at a specific stage of the first meiotic prophase, we performed triple immunofluorescence analysis for DAZL, BOLL and the meiosis marker Synaptonemal Complex Protein 3 (SYCP3; a component of synaptonemal complex which is expressed from pre-meiosis to diplotene in human fetal ovarian germ cells [29]). We detected relatively few SYCP3^+^ germ cells that also expressed DAZL (Figure 4A,B). In contrast, almost all BOLL-positive germ cells also expressed SYCP3 at 14, 15 and 17wga (Figure 4A,B), consistent with BOLL being expressed in germ cells that have reached a later stage of meiosis I than those expressing DAZL.

Quantification of this confirmed the extensive overlap of BOLL and SYCP3 expression (Figure 4C); 69% SYCP3-positive germ cells also expressed BOLL, a fraction significantly higher than the 14% of SYCP3-positive germ cells that also expressed DAZL, 11% of SYCP3-positive germ cells which expressed both DAZL and BOLL, and the 6% of SYCP3-positive germ cells that express neither DAZL or BOLL (SYCP3-positive only; p<0.001). We also compared the extent of co-expression of DAZL and BOLL with another meiosis marker, the phosphorylated isoform of the Ataxia Telangiectasia Mutated (phospho-ATM) protein, which is expressed by germ cells from pre-leptotene to pachytene of meiotic prophase I [30]. Consistent with the pattern seen with SYCP3, we found that a significantly greater proportion of phospho-ATM-positive germ cells also expressed BOLL (78%) than expressed DAZL (37%; p<0.05) (Figure S3A,B). Together, these data suggest that DAZL is expressed before, and persists into, early meiotic prophase I, but is down-regulated around the leptotene/zygotene stage and replaced by BOLL. When the cell reaches the diplotene stage and meiotic arrest and follicle formation are initiated, BOLL expression is extinguished and DAZL expression reactivated.

### The switching of expression from *DAZL* to *BOLL* during fetal oogenesis is not conserved between humans and mice

**Figure 5:**
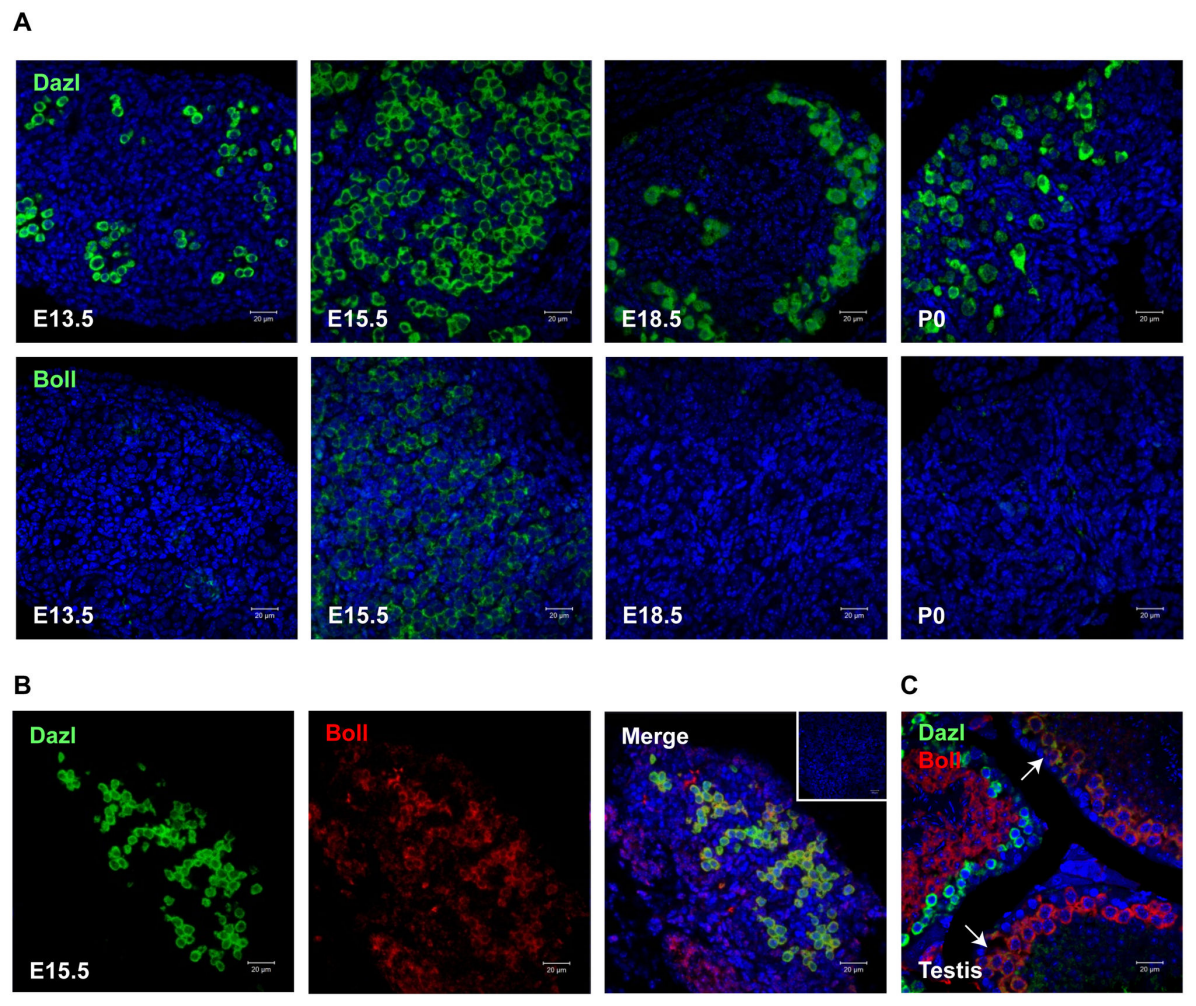
Dazl and Boll proteins display distinct patterns of expression during mouse fetal ovary development. **A**) Single immunostaining for Dazl or Boll in e13.5, e15.5, e18.5 and P0 mouse ovary. Dazl is highly expressed in germ cells through all stages of development, whilst Boll is widely expressed in germ cells at e15.5 but not detectable at earlier or later gestations. **B**) Double immunostaining for Dazl and Boll in e15.5 mouse fetal ovary: Dazl and Boll show extensive co-expression in germ cells at this gestation. **C**) Control showing partial co-localisation of Dazl and Boll in germ cells in the adult mouse testis, an organ known to express both proteins (arrows). Scale bars: 20µm.

Given the striking switch between DAZL and BOLL expression during human fetal ovarian germ cell development, the lack of extensive co-expression of both proteins within the same germ cells, and the apparent lack of an oogenesis defect in Boll-deficient mice, we sought to establish whether the expression patterns of DAZL and BOLL are conserved during fetal oogenesis in humans and mice. We therefore performed single-antigen immunofluorescence to detect either Dazl or Boll on sections of fetal mouse ovaries at embryonic day (e) 13.5, e15.5, e18.5 and postnatal day 0 (P0; Figure 5A), which correspond approximately to meiosis initiation (e13.5), leptotene/zygotene (e15.5), pachytene (e18.5) and diplotene/follicle formation (P0) [31].

Consistent with previous studies, germ cells in the fetal mouse ovary expressed Dazl at all stages examined (Figure 5A). At e13.5, small groups of Dazl-positive germ cells were readily detectable in the fetal mouse ovary, and became more homogeneously distributed throughout the ovary by e15.5. At e18.5 and P0, Dazl-positive germ cells were clearly larger than at previous stages, and predominantly localised towards the periphery of the ovary (Figure 5A). In contrast, Boll was undetectable at e13.5, 18.5 and P0, but readily detectable in large numbers of germ cells at e15.5, with a distribution similar to that of Dazl (Figure 5A).

The distribution of Dazl and Boll in the germ cells of the fetal mouse ovary at e15.5 (Figure 5A) suggested that Dazl and Boll may be extensively co-expressed in the same germ cells during fetal mouse oogenesis. We therefore performed dual-immunofluorescence to detect Dazl and Boll on sections of e15.5 fetal mouse ovaries (Figure 5B). In stark contrast to our findings in the human fetal ovary, we detected extensive co-localisation of Dazl and Boll proteins in the germ cells of the e15.5 fetal mouse ovary. As positive control, we performed dual-immunofluorescence for both proteins on sections of the adult mouse testis (a tissue in which both proteins are known to be expressed), revealing distinct but overlapping patterns of Dazl and Boll expression, with the latter expressed in germ cells at a later stage of development than those expressing Dazl (Figure 5C, far right panel [4,21]). These data reveal the existence of a conserved, transient, window of BOLL expression during fetal oogenesis in both humans and mice, which probably corresponds to a specific stage of meiotic prophase I. However, the striking switch from DAZL expression to BOLL expression during germ cell maturation in the human fetal ovary does not occur in mice.

## Discussion

**Figure 6:**
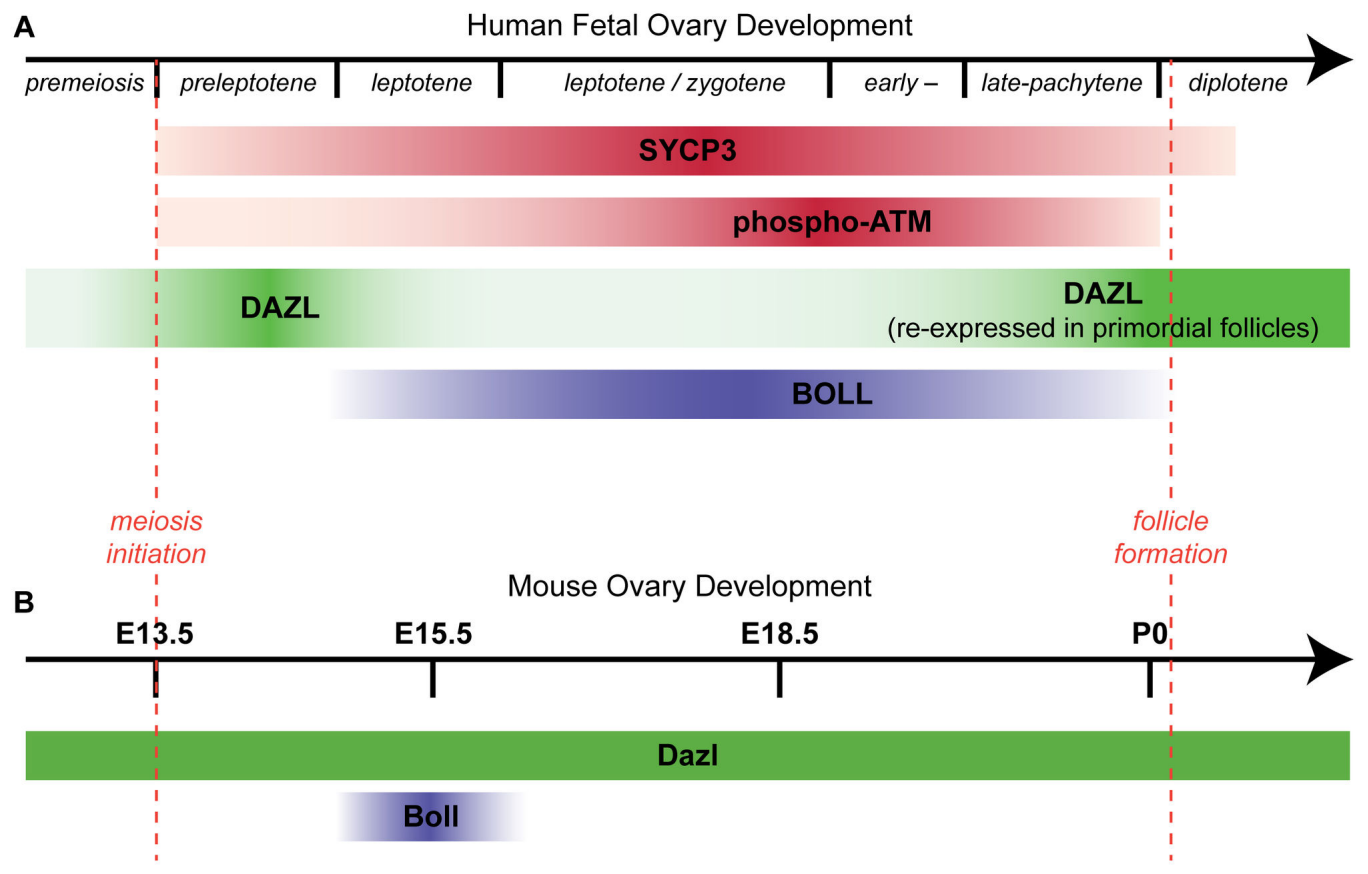
Comparison of human and mouse DAZL/BOLL expression patterns during meiosis. **A**) DAZL and BOLL show distinct spatio-temporal distributions which change during human fetal ovarian development. The timeline is depicted in stages of meiosis in the human due to the wide range of germ cell developmental stages present at any specific week of gestation. DAZL is expressed before and at meiosis initiation but down-regulated afterwards; BOLL is transiently expressed at later stages of meiosis with minimal overlap with DAZL. DAZL is re-expressed in oocytes within primordial follicles. **B**) Expression of Dazl and Boll during meiosis in the feto-neonatal mouse ovary. Dazl is expressed before and throughout meiosis, whereas Boll is detected only transiently expressed around E15.5.

In this report, we have examined the expression and distribution of the RNA-binding proteins DAZL and BOLL during human fetal oogenesis, revealing that whilst *DAZL* and *BOLL* gene expression overlaps using gestational age as the discriminator, DAZL and BOLL proteins are in fact expressed by distinct populations of germ cells at different stages of maturation. We find a progressive pattern of expression as germ cells mature, with DAZL expressed in germ cells prior to, and in the early stages of meiotic prophase I, after which it is down-regulated and BOLL expressed. BOLL in turn is also down-regulated and DAZL re-expressed at the time that germ cells in the human fetal ovary form primordial follicles (Figure 6A). This represents the first demonstration of BOLL protein expression during oogenesis in any mammalian species, and reveals that BOLL is expressed only during a narrow developmental window, which appears to correspond to mid-to-late meiotic prophase I. Finally, we sought to determine whether the switch in expression from DAZL to BOLL that we observe during human fetal oogenesis also occurs during germ cell development in the fetal mouse ovary. We found that although Boll expression in the fetal mouse ovary is also limited to a narrow window during meiotic prophase I (as in humans), Dazl is extensively co-expressed with Boll in the germ cells of the fetal mouse ovary (Figure 6B). Thus in the mouse there is continuous expression of Dazl throughout fetal germ cell development with coincident brief expression of Boll, in contrast to the human fetal ovary, wherein there is a transient loss of DAZL expression at the time of BOLL expression, and the co-expression of both proteins by the same germ cells is rare.

Previous work from our laboratory reported developmental changes in *DAZL* gene expression during human fetal oogenesis, with transcript levels increasing substantially between the first and second trimester [26]. Human ovarian development during the second trimester encompasses two key developmental stages; the formation of germ cell nests and entry into and progression through meiotic prophase I (approximately 12-16wga) and the breakdown of germ cell nests and the assembly of individual oocytes into primordial follicles (approximately 17wga onwards) [32]. The data presented here refines that of Anderson et al. (2007), revealing that the increase in *DAZL* expression occurs between 8-9 and 14-16 weeks gestation, co-incident with the onset of meiosis in the human fetal ovary [33-35]. The expression of DAZL in premeiotic and early meiotic germ cells supports a conserved role for this protein in regulating the entry into meiosis in humans and mice, where Dazl is required in fetal germ cells to enable them to respond appropriately to the meiosis-inducing signal retinoic acid [16]. The expression of *BOLL* overlaps with that of *DAZL*, but the onset of expression is later, with *BOLL* transcripts undetectable in the fetal ovary prior to the onset of meiotic germ cell differentiation. The pattern of overlapping *DAZL* and *BOLL* expression, with the onset of *BOLL* expression occurring slightly later than that of *DAZL*, is very similar to that reported previously in the fetal sheep ovary [24]. In that model, *DAZL* and *BOLL* transcripts are first detected at 38 days post coitum (dpc), but by 49dpc *DAZL* expression is greater than that of *BOLL*, with both genes maximally expressed at 56dpc, corresponding to the early stages of meiotic prophase I. Interestingly, *BOLL* transcript levels, unlike those of *DAZL*, appear to decline slightly between 14-16wga and 17-20wga (although this does not reach statistical significance). This likely reflects a progressive reduction of the number of BOLL-positive germ cells in the fetal ovary at later gestations, as oocytes downregulate BOLL and re-express DAZL as they form primordial follicles, although this may result from a dilution of *BOLL* mRNA due to the emergence of additional BOLL-negative germ cells at this stage.

Although the patterns of *DAZL* and *BOLL* gene expression overlap temporally, our data clearly demonstrate that the spatial distribution of DAZL and BOLL proteins within the human fetal ovary is quite distinct, with DAZL-expressing germ cells towards the periphery, and BOLL-expressing germ cells towards the centre of the ovary. This radial distribution of germ cells reflects a gradient of differentiation, with smaller, less mature germs in the peripheral cortex, and larger, more mature germ less localised in the central medullary region [26,36,37]. Our finding that the nuclear diameter of BOLL-expressing germ cells is greater than that of those expressing DAZL supports this, and demonstrates that BOLL is expressed by germ cells at later stages of development. This is consistent with previous work from our laboratory, which showed that DAZL is expressed in a stage specific fashion during human fetal oogenesis, being downregulated as germ cells mature and begin to express another germ cell-specific RNA binding protein, VASA [26]. The transition from DAZL to VASA expression was also found to occur in the germ cells of the human fetal testis with increasing gestational ages [26], revealing the existence of a germ cell maturation process involving DAZL which is independent of meiosis. The relatively sharp developmental switch from expression of DAZL to expression of BOLL during human fetal oogenesis is distinct from the overlapping patterns of expression of DAZL and VASA, and coupled with the significant correlation of BOLL expression with that of markers of meiosis, suggests the existence of a parallel, meiosis-associated programme of germ cell maturation involving DAZL and BOLL. This is supported by the subsequent downregulation of BOLL, and reactivation of DAZL expression that appears as germ cells form primordial follicles, as follicle formation is dependent on oocytes reaching the diplotene stage of meiotic prophase I and synaptonemal complex disassembly [38]. The data presented here reveals a complex relationship between DAZL, BOLL and meiosis during human fetal ovarian development, and suggests that DAZL may have meiosis-dependent and meiosis-independent roles during human fetal germ cell development, the former being restricted to the fetal ovary and the latter shared with the germ cells of the human fetal testis.

An interesting observation of this study is the identification of a subpopulation of germ cells that express SYCP3, but do not express DAZL or BOLL. It is tempting to speculate that the population of BOLL-negative, DAZL-negative, SYCP3-positive germ cells identified here represents germ cells that are at a stage of development immediately preceding follicle formation, in which BOLL has been downregulated in anticipation of germ cell nest disassembly and follicle formation, but which have not yet proceeded far enough in the follicle assembly process to have reactivated DAZL. However, these germ cells may instead be destined for loss during the extensive waves of apoptosis that occur prior to, and during, follicle formation [32,39,40]. Determining the meiotic stage of the SYCP^+^-only population of germ cells, coupled with assessment of co-expression of SYCP3 with proteins known to be expressed around the time of follicle formation, may provide insight into the developmental stage of these cells.

Zheng et al. (2011) recently reported the construction of a mathematical model of the human fetal ovary, that integrates microarray data on gene expression during human fetal ovarian development with gene ontology and information on protein function, and from this proposed that BOLL may function co-operatively with DAZL to prepare germ cells in the human fetal ovary for entry into meiosis [41]. Whilst the microarray-derived profiles of *DAZL* and *BOLL* gene expression in the human fetal ovary used in their study display similarly overlapping expression to our qPCR data, the immunohistochemical data we present here disputes their model’s prediction, revealing that BOLL is expressed in a different population of germ cells to those expressing DAZL, and the onset of BOLL protein expression occurs only well after meiosis has initiated, precluding extensive physical interaction between the two proteins. Importantly, this underlines the importance of relating transcript data derived from whole organs comprised of multiple different cell types and stages of development to the spatial and temporal distributions of the transcripts and the proteins they encode within the organ.

DAZL and BOLL are RNA-binding proteins, which act to promote the translation of mRNA into protein by binding specific motifs in the 3’ UTRs of target transcripts [5]. Numerous studies have identified putative mRNA targets of DAZL [10,11,42-46], however only a limited number of these have been validated *in vivo* [9,10]. In contrast, little is known about the identity of the mRNA targets of BOLL [47], and whether the motif to which it binds is similar to the GU-rich sequences bound by DAZL remains unclear [48]. The sequential expression of DAZL and BOLL during germ cell maturation in the human fetal ovary, and the co-expression of both proteins in the same germ cells in the fetal mouse ovary around e15.5, raise interesting questions as to the identity of the mRNA targets of these proteins, and to the degree of overlap between them. Do DAZL and BOLL regulate at least a number of the same transcripts, but at different developmental stages in human fetal ovarian germ cell development? Or are their targets distinct, as is might be expected if both proteins are expressed in the same germ cells, as occurs during fetal oogenesis in the mouse? The first possibility indicates that the switch from DAZL to BOLL is largely functional compensation, whereas the latter indicates that the two proteins may have more distinct functions. The comparatively late spermiogenic defect seen in *Boll*-deficient males [4,22] compared with the earlier meiotic and pre-meiotic defects seen in *Dazl*-deficient mice on various genetic backgrounds [1,10,15-18,20,49], supports the latter hypothesis. If this is true, then our data suggest that DAZL and BOLL are required at distinct, relatively non-overlapping developmental stages during human oogenesis, with DAZL required to support the entry into, and progression through the early stages of meiosis (and the formation of primordial follicles), and BOLL required only during established meiotic prophase I. This difference may be useful in identifying the respective mRNA targets of DAZL and BOLL during oogenesis, as comparison of the co-localisation of DAZL, BOLL and the proteins encoded by their putative mRNA targets during human fetal oogenesis will establish which targets co-localise predominantly with one or the other. The capacity of DAZ-family proteins to rescue deficiencies in these genes across diverse species (exemplified by the partial rescue of the spermatogenic defect in *boule-*deficient *Drosophila* by human BOLL [50]) indicates that these proteins can recognise and regulate the translation of the same mRNA targets, raising the possibility that at least some mRNAs targets may be regulated by both DAZL and BOLL at different stages of human fetal oogenesis.

The data presented here raise important questions about the validity of the *Boll*-knockout mouse as a mouse as a model for understanding BOLL function during human female oogenesis. It is tempting to speculate that the simultaneous co-expression of Dazl and Boll in the germ cells of the fetal mouse ovary may present a mechanism by which Dazl could compensate functionally for the absence of Boll, permitting oogenesis to proceed normally in mice. Indeed, such functional redundancy has been proposed as one explanation for the relatively late spermiogenic phenotype seen in *Boll* ^*-/-*^ male mice, which only becomes manifest at a developmental stage after which Dazl is no longer expressed [4]. The sequential expression of DAZL and BOLL by distinct populations of human fetal germ cells at different stages of development precludes the possibility of functional compensation by DAZL in the absence of BOLL during human fetal oogenesis, and raises the intriguing possibility that *BOLL* mutations may contribute to fertility defects in human females.

## Materials and Methods

### Ethics statement

Ethical approval for this study was obtained from Lothian Research Ethics Committee (study code LREC 08/S1101/1). All participants gave informed written consent in accordance with national guidelines. Experiments involving mice were approved by the University of Edinburgh Animal Research Ethics Committee and performed according to the UK Animal (Scientific Procedures) Act 1986.

### Collection of human fetal ovaries

Human fetuses (8-20 weeks gestational age (wga)) were obtained after elective termination of pregnancy. Terminations were for social reasons, and all fetuses used in this study were morphologically normal. Gestational age was determined by ultrasound scan, and confirmed (for second trimester fetuses) by direct measurement of foot length. The sex of the first trimester fetal gonads was determined by PCR for the *SRY* gene [51]. Extra-ovarian tissue was removed from dissected ovaries, which were then either snap frozen on dry ice and stored at -80°C (for subsequent RNA extraction), or fixed in Bouins or 4% Neutral Buffered Formalin (NBF) for 2-3 hours for processing into paraffin blocks for immunohistochemical analysis.

### Animals

C57BL/6 mice were housed on a 12 hour light/dark cycle and fed *ad libitum* according to UK Home Office and local University of Edinburgh ethical standards. The day of vaginal plug detection was designated as embryonic day (e) 0.5. Gonads were isolated from fetuses at e13.5, e15.5 and e18.5, and from neonatal mice on the day of birth (P0), and fixed in 4% NBF for 3 hours before processing into paraffin blocks.

### RNA extraction, cDNA synthesis and qRT-PCR

RNA was extracted from 8-12wga human fetal ovaries using the RNeasy Micro Kit (QIAGEN, Crawley, UK) and from 13-20wga ovaries using the RNeasy Mini Kit (QIAGEN) according the manufacturer’s instructions. cDNA was synthesized using the SuperScript VILO cDNA Synthesis Kit (Life Technologies, Paisley, UK) following manufacturer’s instructions. Identical reactions in which the Reverse Transcriptase (RT) enzyme mix was replaced by nuclease-free dH_2_O were prepared as negative controls.

For measurement of gene expression by qRT-PCR, standard curves for *DAZL, BOLL* and *RPL32* were generated by mixing 1µl serially diluted cDNAs (1/10, 1/25, 1/100, 1/250, 1/1000, 1/2500, 1/10000) with 0.2µl Forward/Reverse primers (25mM stocks), 5µl SYBRGreen Master mix (Life Technologies) and 3.6µl nuclease-free dH_2_O, and run on ABI7900HT thermal cycler (Life Technologies). Ct values were plotted against log concentration, and the resulting slope for each was used to calculate expression within each sample. To permit comparison between individual samples, gene expression was calculated relative to that of the housekeeping gene *RPL32* (the expression of which remains stable across the developmental window examined). The PCR programme used was 50°C for 2 minutes (min), 95°C for 10min, then 40 cycles of 95°C for 15 seconds (sec), 60°C for 1min, and ended with a dissociation stage (95°C for 15sec, 60°C for 15sec and 95°C for 15sec) to check the specificity of PCR products. Quantitation of gene expression in experimental samples was performed using the same conditions as used for standard curves, but diluting the cDNAs 1/10. Results were analysed using ABI SDS2.4 software, Microsoft Excel 2003 and GraphPad Prism 5 software. The sequences of the oligonucleotide primers used in this study can be found in Table S1.

### Immunofluorescence

Human or mouse fetal ovaries were dissected, fixed in Bouins or 4% NBF and processed into wax blocks by standard methods [52]. 5µm sections were cut, mounted on glass slides and dried overnight at 55°C. Slides were dewaxed in xylene (2×5min) and rehydrated through graded alcohols (absolute ethanol: 2×20sec; 90% ethanol: 20sec; 70% ethanol: 20sec). Antigens were retrieved by pressure cooking in 250mL 0.05M pH6 citrate buffer in a Decloaking Chamber (Biocare Medical, CA, USA) set to programme: 125°C for 30sec, cool to 90°C, 10sec. A 30min incubation with 3% hydrogen peroxide diluted in methanol or 10min incubation with Peroxidase Blocking Reagent (DAKO, Glostrup, Denmark) was applied to block endogenous peroxidase action. Tissues were blocked in Phosphate Buffered Saline (PBS (Life Technologies)) containing 20% normal goat or rabbit serum (Diagnostics Scotland, Carluke, UK), the species of serum used being the same as that in which the secondary antibody was raised in) and 5% Bovine Serum Albumin (BSA (Sigma Aldrich, Poole, UK)) or Rodent Block (Mouse on Mouse Polymer IHC Kit (Abcam, Cambridge, UK)) for 30min at room temperature, and then incubated with 100µl primary antibody (see Table S2) per slide in a humidified chamber overnight at 4°C. For negative controls, serum block was applied in place of the primary antibody. Next day, slides were incubated with 100µl peroxidase-conjugated secondary antibody at room temperature for 30min, and then with 50µl fluorescein Tyramide Signal Amplification (TSA (PerkinElmer, Waltham, USA) for 10min. For single antigen detection, tissues were counterstained using 4',6-diamidino-2-phenylindol (DAPI, 1/1000, diluted in PBS) for 10min, mounted and stored at 4°C.

For double-immunofluorescence, the primary antibody to the first antigen was removed by microwaving the slides in 400ml 0.05M citrate buffer for 2.5min, cooled for 1 hour and then blocked in serum block for 30min. The second primary antibody incubation was performed exactly as for the first, as above. On day three, tissues were stained with either peroxidase-conjugated secondary antibody and TSA (as on day 2), or with a fluorophore-conjugated secondary antibody at room temperature for 1h.

For triple-antigen immunofluorescence, the tissues were microwaved and serum blocked again after detection of the second primary antibody, and the third primary antibody incubation performed as for the first and second ones. The signal was detected as on day 3, and finally the tissues were counterstained with DAPI. Images were captured using a 710 Confocal Microscope (Carl Zeiss, Oberkochen, Germany) and Zen 2009 software.

### Germ cell diameter measurement and counting

Tiled images were taken for stained sections using 710 Confocal Microscope (Carl Zeiss) and Zen 2009 software, and different areas include cortex and center of each image were selected randomly for further analysis. For germ cell nuclear diameter measurement, two perpendicular diameters of the germ cell nucleus were measured using Image-Pro Plus (Media Cybernetics, Silver Spring, USA), then averaged and related back to a scale bar to convert pixels to μm. For cell counting, the plugin cell counter of ImageJ (NIH, Maryland, USA) was used and cell numbers were counted manually. The data were analysed using Microsoft Excel 2003 and GraphPad Prism 5 software. Data were analysed using *t* tests, or ANOVA with Neumann-Keuls *post hoc* testing. *P* values less than 0.05 were considered statistically significant.

## Supporting Information

Figure S1 **Validation of anti-DAZL and anti-BOLL antibodies for immunofluorescence.** HEK293 cells were transfected with either pCMV6-DAZL or -BOLL vectors. Anti-DAZL antibodies detected epitopes (green) in pCMV6-DAZL transfected cells, but not in pCMV6-BOLL transfected or untransfected cells (left and centre columns). Mouse anti-BOLL antibody detected epitopes (green) in cells ectopically-expressing BOLL only (right column). Blue: DAPI. Scale bars: 20µm in all panels.
(TIF)

Figure S2 **Specificity of DAZL and BOLL antibodies.** Each blot shows cell lysates of HEK293 cells either mock transfected (transfection agent only) or transfected with vectors expressing hDAZL (pCMV6-hDAZL) or hBOLL (pCMV6-hBOLL). α -tubulin (detected as a band here of ~50kDa-55kDa; predicted molecular weight: 50kDa) was used as loading control. **A**) **and B)** Both anti-DAZL antibodies (mouse and rabbit as indicated) detected a band at ~40-45kDa in only hDAZL-transfected cells (consistent with the predicted molecular weight of DAZL plus the Myc- and FLAG- tags of around 40kDa). **C**) anti-BOLL antibody detected a band of ~39kDa only in hBOLL-transfected cells. No comparable bands were found in mock transfected cells.
(TIF)

Figure S3 **Co-localisation of phospho-ATM with DAZL or BOLL.** **A**) immunofluorescent co-localisation of phospho-ATM with DAZL or BOLL in the human fetal ovary (14 weeks gestation). DAZL shows limited co-expression with phospho-ATM, whereas almost all the phospho-ATM^+^ cells are also BOLL^+^. Arrows indicate germ cells co-expressing of phospho-ATM and DAZL or BOLL, and arrowheads indicate germ cells expressing phospho-ATM only. Scale bars = 20µm. **B**) Quantification of phospho-ATM co-localisation with DAZL and BOLL. BOLL is expressed in ~80% of phospho-ATM^+^ cells, which is significantly higher than the proportion of DAZL/phospho-ATM double positive cells (37%; n=3, 14-16 week human fetal ovaries, *p<0.05).
(TIF)

Methods S1 **This file contains details of the methods used to generate the results in Figures S1-S3, which are not contained within the main body text of the article.**
(DOCX)

Table S1 **Oligonucleotide primers used for qRT-PCR.**
(DOCX)

Table S2 **Antibodies used for Immunofluorescence.**
(DOCX)

Table S3 **Antibodies used for Immunoblotting.**
(DOCX)
