# Correction: A Developmental Stage-Specific Switch from DAZL to BOLL Occurs during Fetal Oogenesis in Humans, but Not Mice

**DOI:** 10.1371/annotation/34304231-e54b-4080-af70-6f957f32d552

**Published:** 2013-11-14

**Authors:** Jing He, Kayleigh Stewart, Hazel L. Kinnell, Richard A. Anderson, Andrew J. Childs

As a result of mistakes in the typesetting process, there were errors in the Figure 4 legend in the PDF. A correct version of the legend is available below.

**Figure 4. BOLL displays greater co-localisation with the meiosis marker SYCP3 than DAZL.**

**A)** Triple immunofluorescence analysis of DAZL (green), BOLL (blue) and SYCP3 (red) in 14 and 17 week ovary. All BOLL^+^ germ cells also express SYCP3 (arrowheads), but only a few DAZL^+^ germ cell express SYCP3 (arrows). Unfilled arrows indicate the SYCP3-positive cells that express neither DAZL nor BOLL. The asterisk indicates a germ cell nest containing BOLL^+^SYCP3^+^, DAZL^+^SYCP3^+^ and DAZL+SYCP- germ cells in close proximity. Scale bars: 20µm. **B)** magnified image of germ cell nest marked with asterisk in A, showing neighboring germ cells expressing different combinations of DAZL, BOLL and SYCP3 expression; arrow denotes DAZL^+^SYCP3^+^ germ cell, arrowhead denotes BOLL^+^SYCP^+^ germ cell. **C)** Quantification of DAZL, BOLL and SYCP3 co-expression. ~69% of SYCP3^+^ cells also express BOLL^+^. This is significantly greater than the percentage of all the other co-expression patterns (n=3 14-17 week human fetal ovaries; ***p<0.001).

