# Correction: A Developmental Stage-Specific Switch from DAZL to BOLL Occurs during Fetal Oogenesis in Humans, but Not Mice

**DOI:** 10.1371/journal.pone.0136009

**Published:** 2015-08-13

**Authors:** Jing He, Kayleigh Stewart, Hazel L. Kinnell, Richard A. Anderson, Andrew J. Childs

In the Funding section, the name of the first funder is incorrect. The correct name is: Medical Research Scotland. The correct funding information is: This work was supported by Medical Research Scotland [grant 354FRG to AJC] and the Medical Research Council [grant G1100357 to RAA]. J.H. is supported by a University of Edinburgh Darwin Scholarship and K.S. is the recipient of a Society for Reproduction and Fertility Summer Vacation Scholarship. The funders had no role in study design, data collection and analysis, decision to publish, or preparation of the manuscript.

## References

[1] He J, Stewart K, Kinnell HL, Anderson RA, Childs AJ (2013) A Developmental Stage-Specific Switch from DAZL to BOLL Occurs during Fetal Oogenesis in Humans, but Not Mice. PLoS ONE 8(9): e73996 doi:10.1371/journal.pone.0073996 24086306 10.1371/journal.pone.0073996 PMC3783425

