# Supplementary figures and images for: A Developmental Stage-Specific Switch from DAZL to BOLL Occurs during Fetal Oogenesis in Humans, but Not Mice
Source: PLoS One. 2013 Sep 25;8(9):e73996. doi: 10.1371/journal.pone.0073996 (PMC3783425; doi:10.1371/journal.pone.0073996)

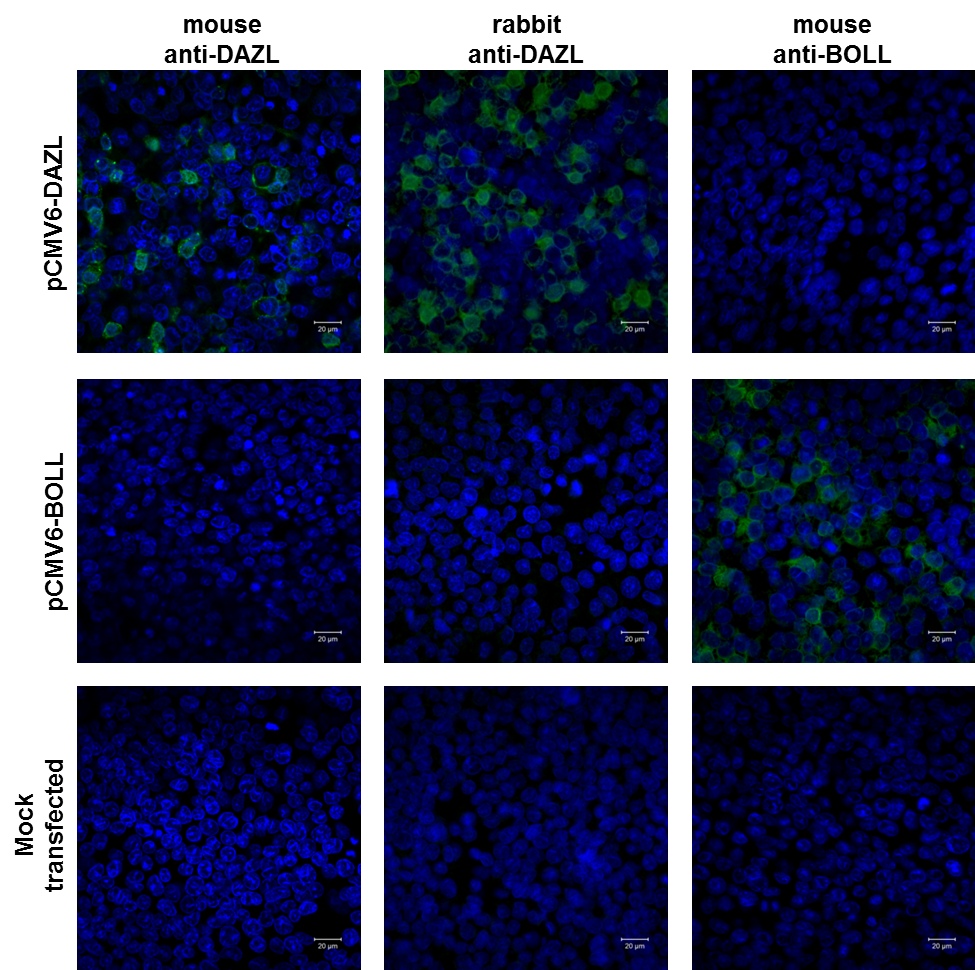

Supplement: Figure S1 — Validation of anti-DAZL and anti-BOLL antibodies for immunofluorescence. HEK293 cells were transfected with either pCMV6-DAZL or -BOLL vectors. Anti-DAZL antibodies detected epitopes (green) in pCMV6-DAZL transfected cells, but not in pCMV6-BOLL transfected or untransfected cells (left and centre columns). Mouse anti-BOLL antibody detected epitopes (green) in cells ectopically-expressing BOLL only (right column). Blue: DAPI. Scale bars: 20µm in all panels. (TIF) [file pone.0073996.s001.tif]

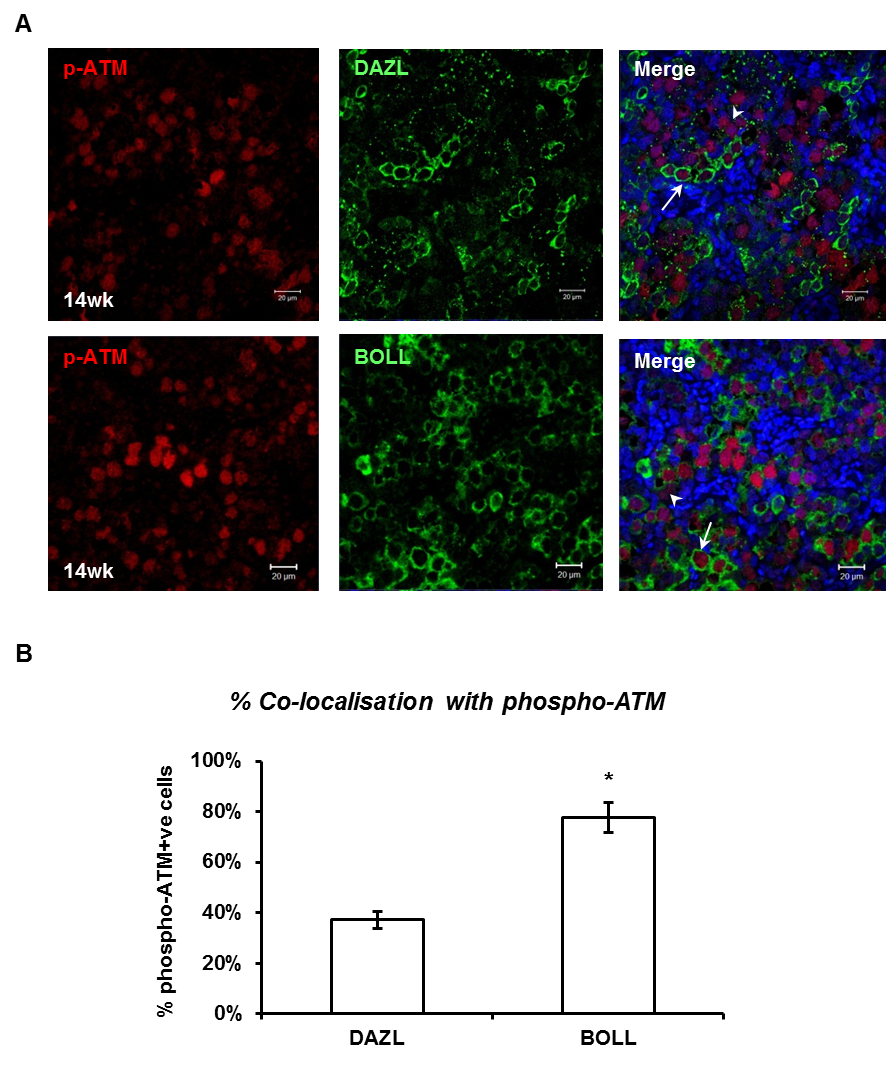

Supplement: Figure S3 — Co-localisation of phospho-ATM with DAZL or BOLL. A) immunofluorescent co-localisation of phospho-ATM with DAZL or BOLL in the human fetal ovary (14 weeks gestation). DAZL shows limited co-expression with phospho-ATM, whereas almost all the phospho-ATM+ cells are also BOLL+. Arrows indicate germ cells co-expressing of phospho-ATM and DAZL or BOLL, and arrowheads indicate germ cells expressing phospho-ATM only. Scale bars = 20µm. B) Quantification of phospho-ATM co-localisation with DAZL and BOLL. BOLL is expressed in ~80% of phospho-ATM+ cells, which is significantly higher than the proportion of DAZL/phospho-ATM double positive cells (37%; n=3, 14-16 week human fetal ovaries, *p<0.05). (TIF) [file pone.0073996.s003.tif]
