# Supplementary material for: A Developmental Stage-Specific Switch from DAZL to BOLL Occurs during Fetal Oogenesis in Humans, but Not Mice
Source: PLoS One. 2013 Sep 25;8(9):e73996. doi: 10.1371/journal.pone.0073996 (PMC3783425; doi:10.1371/journal.pone.0073996)
Supplement: Methods S1 — This file contains details of the methods used to generate the results in Figures S1-S3, which are not contained within the main body text of the article. (DOCX) [file pone.0073996.s004.docx]

**Supplementary Methods for He *et al.,***

**A developmental stage-specific switch from DAZL to BOLL occurs during fetal oogenesis in humans, but not mice**

**Cell culture and transfection**

HEK293 cells were cultured in MEM+GlutaMAX media (Life Technologies, Paisley, UK) containing 10% fetal bovine serum (FBS; Lonza, Basel, Switzerland) at 37°C, 5% CO_2_. Before transfection, HEK293 cells were seeded into 6-well plates and cultured overnight to achieve 50-70% confluency. The media was changed before adding transfection mix. Each transfection mix contained 2.5μg plasmid DNA (either pCMV6-hDAZL or pCMV6-hBOLL (Origene, MD, USA), mixed with 7.5μl TransIT-LT1 Transfection Reagent (Mirus, Madison, USA) and diluted in 250μL Serum-free OPTI-MEM media (Life Technologies). For mock transfection, the mix only contained the transfection reagent and serum-free media. Mixtures were incubated at room temperature for 30 minutes (min) before being added onto the cells. The media was replaced by fresh media after 24 hours (h), and cells were harvested 48h after transfection.

**Fluorescent immunocytochemistry**

HEK293 cells were seeded on 4-well chamber slides (Millipore, Bedford, USA) to achieve 50-70% confluency, and transfected with pCMV6-hDAZL or pCMV6-hBOLL vectors (Origene) as outlined above.), or mock transfected (transfection agent only). 48h after transfection, the cells were washed with ice-cold DPBS (Life Technologies) twice and fixed in ice-cold methanol for 20min, followed by ice-cold and then room temperature DPBS washes, then incubated with Peroxidase Blocking Reagent (DAKO, Glostrup, Denmark) for 10min to block endogenous peroxidase action. The serum blocking along with permeabilization was performed by incubating the cells with PBS (Sigma-Aldrich) containing 20% normal goat serum (Diagnostics Scotland, Carluke, UK), 5% Bovine Serum Albumin (BSA; Sigma-Aldrich, Poole, UK) and 0.2% IGEPAL-CA630 (Sigma-Aldrich) for 30min. Then the mouse anti-DAZL (1/100, AbD Serotec, Oxford, UK), rabbit anti-DAZL (1/1000, Cell Signalling Technologies, Hitchin, UK) or mouse anti-BOLL (1/200, Abcam, Cambridge, UK) primary antibodies were applied on the cells and incubated overnight at 4°C. Next day, the cells were incubated with different secondary antibodies (as detailed in Table S2), and 10min fluorescein Tyramide Signal Amplification (TSA; PerkinElmer, Waltham, USA) staining was performed on the cells. The cells were finally counterstained with DAPI and images were captured using a 710 Confocal Microscope (Carl Zeiss, Oberkochen, Germany) and Zen 2009 software.

**Protein extraction and western blotting**

HEK293 cells were washed in ice-cold Dulbecco's Phosphate-Buffered Saline (DPBS; Life Technologies) twice, and lysed with 100μl Radio ImmunoPrecipitation Assay (RIPA) buffer (50mM Tris-Cl ph7.4, 150mM NaCl, 1% NP40, 0.25% sodium deoxycholate, plus 1 Roche Complete Mini Protease inhibitor cocktail tablet (Roche, Penzberg, Germany)). The protein concentration was determined by using Bio-Rad DC Protein Assay Reagent (Bio-Rad, Richmond, USA) at absorbance 620nm.

For SDS-PAGE electrophoresis, 20μg lysate was mixed with 4μl Loading Buffer (625mM Tris pH6.8, 5% Glycerol, 2% SDS, 0.0025% Bromophenol Blue, 2.5% β-mercaptoethanol), and extra RIPA buffer was added to a final volume of 20μL. The mix was boiled for 5min and either separated on 4-20% Precise Protein Gels (Thermo-Scientific, Waltham, USA) using an XCell SureLock Mini-Cell tank (Invitrogen), with 1X Tris-HEPES SDS running buffer (Thermo-Scientific) at 120V, or on 12% Ready Gel Tris-HCl Gels (Bio-Rad) using Mini-PROTEAN Tetra Cell tank (Bio-Rad), with 1X Tris-Glycine SDS running buffer (25mM Tris, 250mM Glycine and 0.1% SDS) at 200V. 6μL PageRuler Prestained Protein Ladder (Fermentas/Thermo-Scientific) was used to determine protein sizes.

Proteins were transferred onto PVDF membranes (Immobilon-FL Transfer membrane, Millipore Corporation, Bedford, USA) using a Pierce Fast Semi-Dry Blotter System (Pierce/Thermo-Scientific) at 25V for 7min with 1X Semi-Dry Transfer Buffer (Thermo-Scientific). After transfer, blots were stained with Ponceau S (Sigma-Aldrich) to check the transfer efficiency, and blocked in Blok-FL Buffer (Millipore) for 1 hour, followed by incubation with primary antibodies (see Supplementary Table 3) at 4°C overnight. α-tubulin (Sigma-Aldrich) was detected as loading control. Next day, blots were incubated in fluorophore-conjugated secondary antibody (diluted 1/10000 in blocking buffer; Table S3) for 1 hour at room temperature, and finally scanned on Li-Cor System (Li-Cor, Lincoln, USA) and using Odyssey V3.0 software.
