# Supplementary material for: A Developmental Stage-Specific Switch from DAZL to BOLL Occurs during Fetal Oogenesis in Humans, but Not Mice
Source: PLoS One. 2013 Sep 25;8(9):e73996. doi: 10.1371/journal.pone.0073996 (PMC3783425; doi:10.1371/journal.pone.0073996)
Supplement: Table S1 — Oligonucleotide primers used for qRT-PCR. (DOCX) [file pone.0073996.s005.docx]

**Table S1: Oligonucleotide primers used for qRT-PCR:**

| **Gene** | **Forward (5'→3')** | **Reverse (5'→3')** |
| --- | --- | --- |
| *RPL32* | CATCTCCTTCTCGGCATCA | AACCCTGTTGTCAATGCCTC |
| *DAZL* | GAAGGCAAAATCATGCCAAACAC | CTTCTGCACATCCACGTCATTA |
| *BOLL* | TATAAGGATAAGAAGCTGAACATTGGT | CGAAGTTACCTCTGGAGTATGAAAATA |
