# Supplementary material for: A Developmental Stage-Specific Switch from DAZL to BOLL Occurs during Fetal Oogenesis in Humans, but Not Mice
Source: PLoS One. 2013 Sep 25;8(9):e73996. doi: 10.1371/journal.pone.0073996 (PMC3783425; doi:10.1371/journal.pone.0073996)
Supplement: Table S2 — Antibodies used for Immunofluorescence. (DOCX) [file pone.0073996.s006.docx]

**Table S2: Antibodies used for Immunofluorescence**

| Primary Antibody | Dilution | Species Raised | Secondary antibody | Manufacturer |
| --- | --- | --- | --- | --- |
| DAZL | 1/100 | mouse | Goat anti-Mouse Alexa 488 | AbD Serotec |
|  | 1/500 | mouse | Goat anti-Mouse peroxidase fab | AbD Serotec |
|  | 1/500 | rabbit | Goat anti-Rabbit peroxidase | Cell Signalling |
| BOLL | 1/50 (old)  1/200 (new) | mouse | Goat anti-Mouse peroxidase fab or Mouse-on-Mouse HRP polymer | Abcam |
| SYCP3 | 1/50000 | rabbit | Goat anti-Rabbit peroxidase | Abcam |
| phospho-ATM | 1/200 | mouse | Rabbit anti-Mouse peroxidase fab | Rockland |
