# Supplementary material for: A Developmental Stage-Specific Switch from DAZL to BOLL Occurs during Fetal Oogenesis in Humans, but Not Mice
Source: PLoS One. 2013 Sep 25;8(9):e73996. doi: 10.1371/journal.pone.0073996 (PMC3783425; doi:10.1371/journal.pone.0073996)
Supplement: Table S3 — Antibodies used for Immunoblotting. (DOCX) [file pone.0073996.s007.docx]

**Table S3: Antibodies used for Immunoblotting:**

| Primary Antibody | Dilution | Species Raised | Secondary antibody | Manufacturer |
| --- | --- | --- | --- | --- |
| DAZL | 1/200 | mouse | Donkey anti-Mouse 800 | AbD Serotec |
| DAZL | 1/1000 | rabbit | Donkey anti-Rabbit 600 | Cell Signalling |
| BOLL | 1/100 | mouse | Donkey anti-Mouse 800 | Abcam |
| α-tubulin | 1/5000 | mouse | Donkey anti-Mouse 800 | Sigma-Aldrich |
